# Immobilization and Monitoring of *Clostridium carboxidivorans* and *Clostridium kluyveri* in Synthetic Biofilms

**DOI:** 10.3390/microorganisms13020387

**Published:** 2025-02-10

**Authors:** Josha Herzog, Anna C. Jäkel, Friedrich C. Simmel, Dirk Weuster-Botz

**Affiliations:** 1 Biochemical Engineering, Department of Energy and Process Engineering, TUM School of Engineering and Design, Technical University of Munich, Boltzmannstraße 15, D-85748 Garching, Germany; josha.herzog@tum.de; 2 Physics of Synthetic Biological Systems, Department of Bioscience, TUM School of Natural Sciences, Technical University of Munich, Am Coulombwall 4a, D-85748 Garching, Germany; anna.jaekel@tum.de (A.C.J.); simmel@tum.de (F.C.S.)

**Keywords:** clostridia, cell immobilization, bioproduction, co-culture

## Abstract

The growing need for sustainable biotechnological solutions to address environmental challenges, such as climate change and resource depletion, has intensified interest in microbial-based production systems. Synthetic biofilms, which mimic natural microbial consortia, offer a promising platform for optimizing complex metabolic processes that can convert renewable feedstocks into valuable chemicals. In this context, understanding and harnessing the interactions between co-immobilized microorganisms are critical for advancing bioprocesses that contribute to circular bioeconomy goals. In this study, we investigated the viability and metabolic activity of *Clostridium carboxidivorans* and *Clostridium kluyveri* within a synthetic, dual-layered biofilm composed of agar hydrogel. This setup compartmentalized each bacterial species. Embedding the bacteria in a structured biofilm offers numerous opportunities for bioproduction, but the inability to monitor cell growth or movement within the immobilization matrix limits process insights. To address this, we adapted a fluorescence in situ hybridization (FISH) protocol, enabling precise, species-specific visualization of bacterial distribution and growth within the gel matrix. Batch processes with the dual-layered biofilm in anaerobic flasks, designed with a metabolic advantage for *C. kluyveri,* revealed distinct growth dynamics. *C. kluyveri* exhibited significant metabolic activity, forming clusters at low initial cell concentrations and converting ethanol and acetate into 1-butyrate and 1-hexanoate, indicating viability and cell growth. *C. carboxidivorans* remained evenly distributed without significant growth or product formation, suggesting that while the cells were viable, they were not metabolically active under the experimental conditions. Both bacterial species were confined to their respective compartments throughout the process, with *C. kluyveri* showing enhanced substrate conversion at higher initial cell densities in the hydrogel. The pH drop throughout the batch experiment likely contributed to incomplete substrate consumption, particularly for *C. kluyveri*, which thrives within a narrow pH range. These findings highlight synthetic biofilms as a promising platform for optimizing microbial interactions and improving bioprocess efficiency, especially in applications involving complex metabolic exchanges between co-immobilized microorganisms. Further research will focus on applying conditions to support the growth and metabolic activity of *C. carboxidivorans* to explore spatial dynamics of bacterial migration and cooperative relationships in the synthetic biofilm.

## 1. Introduction

Climate change has increased the attention given to developing production processes that rely on renewable resources. A promising strategy for generating organic chemicals involves the gasification of organic waste materials to produce synthesis gas, which primarily consists of nitrogen (N_2_), carbon dioxide (CO_2_), carbon monoxide (CO), and hydrogen (H_2_) [1,2]. Acetogenic microorganisms can utilize the synthesis gas components CO_2_ with H_2_ or CO, effectively transforming greenhouse gases into valuable multi-carbon organic compounds [3,4]. These biological CO_2_ fixation processes not only mitigate greenhouse gas emissions but also contribute to the creation of a sustainable circular carbon economy by converting industrial emissions into useful chemical products [5].

In addition to the gasification of organic waste materials, synthesis gas, or syngas, is often generated as a by-product of industrial activities [6,7]. Anaerobic and acetogenic microorganisms have the unique ability to convert syngas into organic acids and alcohols. These compounds can serve as energy carriers or as raw materials in various industrial applications. Acetogenic bacteria are capable of converting CO_2_ and H_2_ autotrophically into chemicals like acetate, ethanol, 1-butyrate, 1-butanol, and others with high energetic efficiency, reaching up to 70–90% [8].

Genetic engineering efforts have expanded the variety of chemicals that acetogens can produce, resulting in engineered strains that create acetone, isopropanol, 3-hydroxypropionate, mevalonate, isoprene, and other chemicals from syngas. However, the reported yields and concentrations of these products remain relatively low [9].

Among acetogenic bacteria, *Clostridium carboxidivorans* has been extensively studied for its ability to synthesize small amounts of medium-chain fatty acids like 1-butyrate and 1-hexanoate and the corresponding alcohols, besides its ability to produce greater amounts of acetate and ethanol [10,11]. Since those acetogenic microorganisms that utilize H_2_/CO_2_ or CO are typically only capable of producing short-chain organic compounds in significant quantities, combining them with other anaerobic microorganisms presents a promising approach [12]. In a synthetic co-culture within a stirred tank bioreactor, these combined microorganisms can generate longer-chain fatty acids and alcohols. Microorganisms such as *Clostridium kluyveri* are particularly suitable for this purpose, as they are capable of converting short-chain products into more complex, valuable compounds [12,13,14].

*C. kluyveri* can transform a mixture of acetate and ethanol anaerobically into 1-butyrate, 1-hexanoate [15], and trace amounts of 1-octanoate [16]. Ethanol is initially oxidized by *C. kluyveri* to acetyl-CoA, which then reacts with another acetyl-CoA molecule to form acetoacetyl-CoA. Through a series of reactions resembling reverse β-oxidation, butyryl-CoA is produced. This compound combines with acetate, resulting in the release of 1-butyrate and acetyl-CoA. When butyryl-CoA continues through the cycle, it eventually leads to the formation of hexanoyl-CoA, which exits the cycle as 1-hexanoate along with acetyl-CoA. During these chain elongation steps, the Rnf complex facilitates energy conservation from reduced ferredoxin by utilizing the exothermic reduction of intermediates, such as crotonyl-CoA and 2-hexenoyl-CoA, into butyryl-CoA or carbonyl-CoA [15,17,18,19].

*C. kluyveri* has been used multiple times in co-cultures with acetogenic bacteria as a chain elongator and has shown promising results [20,21,22,23]. In co-cultures with *C. carboxidivorans*, *C. kluyveri* has also been successfully applied [24,25]. It has been observed that at 800 mbar CO and pH 6.0, chain elongation activity occurred, although the growth of *C. kluyveri* was limited [25]. Organic acids produced by *C. kluyveri* were converted by *C. carboxidivorans* into 1-butanol and 1-hexanol, resulting in a threefold increase in the 1-butanol concentration and enabling 1-hexanol production compared to a *C. carboxidivorans* mono-culture [25]. At 100 mbar CO, *C. kluyveri* showed better growth, but *C. carboxidivorans* had a reduced ability to produce alcohols, while the accumulation of organic acids led to a steady decline in the *C. carboxidivorans* population [25]. Thus, a synthetic co-culture involving *C. carboxidivorans*, which produces acetate, ethanol, 1-butyrate, 1-butanol, 1-hexanoate, and 1-hexanol from CO, alongside *C. kluyveri*, which converts acetate and ethanol into 1-butyrate and 1-hexanoate, presents a promising strategy. The 1-butyrate and 1-hexanoate can then be directly reduced by *C. carboxidivorans* to 1-butanol and 1-hexanol, making this co-culture an effective approach for broadening the product spectrum of syngas fermentation but still, like with co-cultures of acetogenic and chain elongating bacteria, facing some challenges.

Challenges associated with those co-cultures often arise from the suboptimal growth of one of the microorganisms involved. In the example of *C. carboxidivorans* and *C. kluyveri*, low CO levels are beneficial for *C. kluyveri* as their growth experiences less inhibition [26], but this condition is detrimental to *C. carboxidivorans* due to a reduced substrate availability [25]. Conversely, high CO concentrations hinder the growth of *C. kluyveri*, while providing ample substrate for *C. carboxidivorans* [26]. Therefore, it is prudent to consider cell immobilization techniques, which could spatially separate the cells within different milieus and also allow for growth decoupled production.

Research on the self-immobilization of anaerobic microorganisms colonizing surfaces has yielded significant advancements in various applications, highlighting the potential for enhanced biotechnological processes [27]. For instance, Weuster-Botz (1993) [28] and Weuster-Botz et al. (1993) [29] demonstrated advanced methods for heterotrophic ethanol production using *Zymomonas mobilis* self-immobilized in macroporous glass beads within fluidized bed biofilm reactors. By developing kinetic models and employing innovative colonization techniques, both research efforts achieved high ethanol yields and contamination resistance, even under unsterile conditions. The systems provided stable long-term operation, improved space–time yields, and reduced residence times, showcasing significant advantages for economical and efficient bioethanol production. An example of autotrophic self-colonization of macroporous glass carriers was reported by Riegler et al. (2019), showing continuous conversion of CO_2_ and H_2_ using *Clostridium aceticum* in biofilm reactors, illustrating the effectiveness of immobilized systems for sustained biochemical production [30].

Natural biofilm formation by the microorganisms themselves is time-consuming, especially under autotrophic conditions, if it works at all. Applying synthetic biofilms, e.g., by mixing the cells in a hydrogel followed by distribution of the liquid hydrogel on surfaces before crosslinking to form a synthetic biofilm matrix with the embedded cells, may be a solution. *Shewanella oneidensis* has been successfully immobilized in an agarose hydrogel, demonstrating the feasibility of this approach for bioprocessing [31].

While *C. kluyveri* has not yet been explicitly immobilized in hydrogels, it has been successfully utilized in conjunction with wheat straw for the production of hexanoate [32]. *C. carboxidivorans* has also been studied in biofilm reactors, with multiple studies highlighting its application in syngas fermentation [33,34,35]. Furthermore, Kim et al. (2018) introduced a novel synthetic co-culture involving *Clostridium* spp. and *Megasphaera hexanoica* using submerged hollow-fiber membrane bioreactors for caproic acid production [36]. Shen et al. (2018) demonstrated the conversion of syngas (CO and H_2_) to biochemicals through mixed culture fermentation in mesophilic and thermophilic hollow-fiber membrane biofilm reactors, while Wang et al. (2018) explored mixed culture fermentation of synthesis gas in microfiltration and ultrafiltration hollow-fiber membrane biofilm reactors [37,38]. In another significant study, Steger et al. (2017) optimized continuous gas fermentation by immobilizing acetate-producing *Acetobacterium woodii*, showcasing the advantages of immobilization in enhancing fermentation efficiency [39]. Lastly, Thanapornsins et al. (2024) presented a novel approach for butanol fermentation from sweet sorghum stem juice using a co-culture of *Arthrobacter* sp. and immobilized *Clostridium beijerinckii* in 1 L-bottles and 5 L- and 30 L-stirred-tank bioreactors. Collectively, these studies underscore the progress made in the immobilization of anaerobic microorganisms and their application in biotechnological processes, paving the way for further research and innovation in this field [40].

To optimize biological processes, it is essential to have extensive knowledge of as many process variables as possible. The conventional method for determining cell dry weight (CDW) concentrations over time, based on photometric measurement of optical density, is only applicable to suspended cells. For immobilized cells, the most common non-invasive methods are bacterial cell morphometry, Raman microscopy, confocal laser scanning microscopy, optical coherence tomography, and combined methods [41,42]. However, in co-cultures, it is necessary to differentiate between the individual cells, which are visually indistinguishable. In suspension, this issue was addressed by Bäumler et al. (2021) and Schneider et al. (2021) for *Clostridium carboxidivorans* and *Clostridium kluyveri* in co-culture using fluorescence in situ hybridization (FISH) and analysis with flow cytometry [24,25].

FISH was introduced by Gall and Pardue in 1969 as a molecular technique for detecting and locating specific nucleic acid sequences within intact cells [43]. Initially, FISH employed radioactive labels, but advancements by Rudkin and Stollar in 1977 introduced fluorescent labeling, offering improved safety and resolution through immunofluorescence [44]. In FISH, fluorescently labeled DNA or RNA probes bind to target RNA sequences, such as the 16S or 23S ribosomal RNA (rRNA). The 16S rRNA gene is a highly conserved component of the small subunit of prokaryotic ribosomes and plays a critical role in protein synthesis. The same is true for the 23S rRNA which is an important part of the big subunit of the prokaryotic ribosomes. Despite their conserved regions, they also contain hypervariable regions that are unique to each bacterial species, making it an ideal target for identifying and distinguishing bacteria at the species level [45]. By targeting these variable regions of the 16S or 23S rRNA, FISH enables precise identification and spatial localization of specific bacteria within complex microbial communities. This technique has become a valuable tool in microbiology and biotechnology, allowing researchers to identify and differentiate bacterial populations that would otherwise appear indistinguishable under conventional microscopy. Additionally, FISH has been adapted for advanced applications, such as sorting labelled cells to separate living bacteria post-labelling, a method particularly useful for microbial studies and aligned closely with our research goals [46].

To date, there has been no research involving the combination of *C. carboxidivorans* and *C. kluyveri* in a synthetic biofilm. This study aims to determine whether these anaerobic microorganisms remain metabolically active and capable of growth when immobilized in space in an agar hydrogel matrix. Building on the established FISH techniques for suspended *C. carboxidivorans* and *C. kluyveri* [24], our research will adapt FISH to identify immobilized cells within a synthetic biofilm. This adaptation is essential for precise quantification of varying species, enabling a more detailed understanding of the co-culture’s metabolic interactions and biotechnological potential. Building on this, this study aims to develop a method for quantitative determination of individual CDW concentrations in synthetic biofilms over the course of a batch process, utilizing FISH labelling and subsequent analysis through fluorescence microscopy.

## 2. Materials and Methods

### 2.1. Microorganisms and Growth Conditions

The bacterial strains used in this study were *Clostridium kluyveri* DSM 555 and *Clostridium carboxidivorans* DSM 15243, obtained from the German Collection of Microorganisms and Cell Cultures (DSMZ, Braunschweig, Germany). Anaerobic cultivation was performed at 37 °C in 500 mL anaerobic flasks sealed with butyl rubber stoppers (Glasgerätebau Ochs, Bovenden, Germany) under an atmosphere of 100% N_2_ for precultures. The atmosphere used for the synthetic biofilm studies is described in Section 2.4. The medium used for cultivation was a modified Hurst medium [47], prepared by supplementing the original formulation with a buffer (15.0 g L^−1^ MES for *C. carboxidivorans* and 2.5 g L^−1^ sodium bicarbonate for *C. kluyveri*) and a reducing agent (L-cysteine-HCl 2 H_2_O, 1.0 g L^−1^ for *C. carboxidivorans*, and 0.4 g L^−1^ for *C. kluyveri*), following the protocol described by Schneider et al. (2021) [24]. The medium was further supplemented with different carbon sources according to the specific requirements of the microorganisms: 5.0 g L^−1^ glucose was used for the precultures of *C. carboxidivorans*, and *C. kluyveri* was cultivated with 10 g L^−1^ potassium acetate and 20 mL L^−1^ ethanol. The pH was adjusted to pH 6.0 for *C. carboxidivorans* and to pH 6.8 for *C. kluyveri*. The medium was prepared anaerobically as described in the literature [48,49]. For an overview, refer to the Supplementary Information Table S1.

### 2.2. Preculture

Strains obtained from the DSMZ (Braunschweig, Germany) were initially cultivated in modified Hurst medium, after which glycerol was added, and the cultures were stored at −80 °C as frozen stocks until required for preculture preparation. For the preparation of precultures, 2.5 mL of the frozen stock culture was thawed and inoculated into anaerobic flasks with a nitrogen-filled headspace at a total pressure of 1.0 bar. Inoculation was conducted using a syringe (BD Discardit II, Becton Dickinson, Franklin Lakes, NJ, USA) and sterile needles (Sterican 0.9 × 70 mm, B. Braun, Melsungen, Germany) through a butyl rubber stopper (Glasgerätebau Ochs, Bovenden, Germany).

*C. carboxidivorans* and *C. kluyveri* were cultivated heterotrophically in 500 mL anaerobic flasks containing 100 mL of modified Hurst medium, supplemented with 5.0 g L^−1^ glucose for *C. carboxidivorans* and 10 g L^−1^ potassium acetate and 20 mL L^−1^ ethanol for *C. kluyveri* as the carbon source. For *C. kluyveri*, an additional amount of 2.5 g L^−1^ Na_2_HCO_3_ was added to supply bicarbonate/carbon dioxide as an inorganic carbon source [50,51]. 0.5 g L^−1^ cysteine-HCl was added for both organisms as a reducing agent. The cultures were incubated at 37 °C in a shaking incubator (Wisecube WIS-20R, witeg Labortechnik GmbH, Wertheim, Germany) at 100 rpm with a 2.5 cm eccentricity for 24 h for *C. carboxidivorans* and for 120 h for *C. kluyveri*.

Cell harvesting was performed during the exponential growth phase by centrifugation at 3620 rcf for 10 min (Hettich Zentrifuge, Rotina 50 RS, Hettich GmbH, Tuttlingen, Germany). The harvested cells were then resuspended in anaerobic phosphate-buffered saline (PBS, pH 7.4) for inoculation into the hydrogel.

### 2.3. Preparation of Synthetic Biofilms

The hydrogel was prepared by dissolving 18 g L^−1^ agar-agar in the modified Hurst medium described above through autoclaving at 121 °C for 20 min. To create the synthetic biofilm, the hydrogel was cooled to room temperature under a nitrogen atmosphere. Once the temperature reached 45 °C, the hydrogel was inoculated with the previously prepared cell suspension in PBS. Defined volumes of the inoculated hydrogel were then dispensed into molds or anaerobic flasks using a positive displacement pipette (Transferpettor Digital 2000–10,000 µL; BRAND GMBH + CO KG, Wertheim, Germany), depending on the experimental requirements. The molds or flasks were left to cool to room temperature under a nitrogen atmosphere, forming the synthetic biofilm.

### 2.4. Co-Cultivation in Anaerobic Flasks with Synthetic Biofilms

Co-cultures in 500 mL anaerobic flasks were prepared by first applying a 10 mL layer of the first synthetic biofilm to the bottom of the flasks, following the procedure described above followed by the addition of a second 10 mL layer of synthetic biofilm applied after the first layer had solidified, resulting in biofilms with an average thickness of 4 mm. The biofilm layers were then overlaid with 100 mL of the appropriate medium under a nitrogen atmosphere. The flasks were sealed with a butyl rubber stopper (Glasgerätebau Ochs, Bovenden, Germany), and the gas atmosphere inside the flasks was replaced with the designated gas mixture using sterile needles (Sterican 0.9 × 70 mm, B. Braun, Melsungen, Germany) through the butyl rubber stopper.

The co-culture experiments in synthetic biofilms were carried out in triplicates and conducted at 37 °C for 7 days, with conditions favoring the metabolism of *C. kluyveri* to enhance its growth and product formation. The modified Hurst medium, as described above, was adjusted to pH 6.8 and supplemented with 10 g L^−1^ potassium acetate, 20 mL L^−1^ ethanol, 2.5 g L^−1^ sodium bicarbonate, and 1.0 g L^−1^ L-cysteine-HCl 2H_2_O. The gas atmosphere within the system was maintained at a 1:9 N_2_:CO_2_ ratio with a total pressure of 1.5 bar.

Samples for FISH analysis to assess cell distribution within the synthetic biofilms were taken at both the start and the end of the process. To generate data on cell distribution during the process as well, additional experiments (Section 3.4) were conducted in which anaerobic flasks were terminated at 24 h intervals, and a gel sample was collected in the same manner. Further processing of the gel samples for FISH analysis is detailed in Section 2.6. Detachment of cells from the synthetic biofilm was periodically assessed over a period of up to 7 days by measuring the optical density at 600 nm in the liquid phase using a spectrophotometer (Genesys 10S UV-Vis; Thermo Scientific, Neuss, Germany). The organic acid and alcohol concentrations in the culture supernatant were measured via high-performance liquid chromatography (HPLC). To perform these measurements, 2 mL samples were aseptically collected from the anaerobic flasks using sterile, single-use syringes (BD Discardit II, Becton Dickinson, Franklin Lakes, NJ, USA) and sterile needles (BD Microlance 3, 0.7 × 30 mm, Becton Dickinson, Franklin Lakes, NJ, USA).

### 2.5. High-Performance Liquid Chromatography (HPLC)

Organic acids and alcohols were quantified using HPLC (1100 Series, Agilent Technologies, Santa Clara, CA, USA), which was equipped with a refractive index (RI) detector and an Aminex HPX-87H ion exchange column (Bio-Rad, Munich, Germany). The separation was achieved using 5 mM H_2_SO_4_ as the mobile phase at a constant flow rate of 0.6 mL min^−1^, with the column maintained at 60 °C. Before injection into the HPLC system, samples were filtered through a 0.2 µm cellulose filter (Chromafil RC20/15 MS; Macherey-Nagel GmbH & Co. KG, Düren, Germany).

### 2.6. Monitoring of Immobilized Bacteria Using FISH

**Figure 1:**
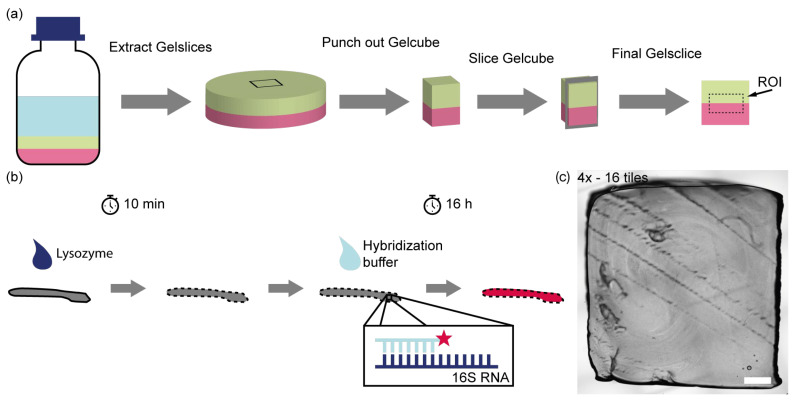
Preparation of gel slices to monitor the growth of bacteria. (**a**) Gel cubes are extracted from the bottle cultures with an edge length of 10 mm with a custom-made polylactide (PLA) punch. These cubes were cut into 1 mm-thick slices with a microtome so that the interface between the two different bacteria cultures could be observed. The figure shows the region of interest (ROI) which is later observed with a microscope. (**b**) To visualize the different types of bacteria, they are stained with FISH probes. The membrane of the bacteria was penetrated with lysozyme to make it permeable for the FISH probes, which were designed to bind to specific 16S or 23S rRNA of the two types of bacteria. The probes were functionalized with a fluorescent dye (red star) at the 5 prime end to measure the intensities with a confocal microscope. (**c**) Gel slices with a 10 mm-long edge length were cut; scalebar: 1000 µm.

The procedure for monitoring the growth of immobilized bacteria within the synthetic biofilm, using fluorescence in situ hybridization (FISH) and confocal microscopy, is outlined in Figure 1. Gel slices were carefully prepared from the bottle cultures to enable observation of bacterial growth at the interface between the two species. Initially, gel cubes, each with an edge length of 10 mm, were punched out from the anaerobic bottles (SI—Figure S2) using a 3D-printed custom-made PLA punch (SI—Figure S3) which could be connected with a motoric pipette filler (Pipetus, Hirschmann, Eberstadt, Germany). These cubes were then precisely sliced into 1 mm-thick sections (SI—Figure S5) using a microtome with a razor blade (Classic, Wilkinson Sword, High Wycombe, UK) (SI—Figure S4a), ensuring the bacterial interface was preserved.

To differentiate between the two bacterial types, FISH was employed, utilizing probes designed to bind specifically to the 23S rRNA of each species. Prior to probe application, the bacterial membranes were treated with lysozyme to increase permeability, allowing the probes to penetrate the cells. The FISH probes, functionalized with fluorescent dyes at the 5′ end, enabled visualization of the bacterial species under a confocal microscope by measuring fluorescence intensity. A universal FISH probe, EUB338 [52], was modified with the fluorescent dye Alexa 488 to stain both bacteria species as a positive control. To distinguish the two bacterial species, FISH probes that specifically bind either *C. carboxidivorans* or *C. kluyveri* were used. The probes ClosCarb_1516 and ClosKluy_1516 were modified with the fluorescent dyes Cy 3 and Cy5, respectively. As shown in Figure 1c, the prepared gel slices retained their 10 mm edge length, with the scale bar indicating 1000 µm for reference.

This method, adapted from Schneider et al. (2021) [24], was validated through preliminary tests, which confirmed its efficacy (see Supporting Information Figure S1). Subsequently, this approach was employed to analyze bacterial growth patterns and spatial distribution within the synthetic biofilm, allowing for precise monitoring of the co-cultivated species.

### 2.7. FISH Probes

To stain the bacteria, a general FISH probe “EUB338” that targets the 16S rRNA of most bacteria strains was used [52]. To differentiate the two cultures, a specific 23S rRNA oligonucleotide probe for *C. carboxidivorans* and *C. kluyveri* were used. The sequences were developed by Schneider et al. (2021) [24]. The probes were ordered from Integrated DNA Technologies, B.V. (IDT, Leuven, Belgium). They were synthesized with a fluorescent dye at the 5 prime end and purified with HPLC. To be able to image all three probes at the same time, the orthogonal fluorescent dyes Alexa488, Cy3, and Cy5 were used to label the probes (Table 1).

### 2.8. Gel Bacteria Staining

The gel slices with bacteria were placed in a 24-well plate and dehydrated sequentially with 80% ethanol followed by 99% ethanol, each for 3 min, removing the solution after each dehydration step. Cell lysis was performed by adding 800 µL of PBS and 200 µL of lysozyme solution (10 mg mL^−1^) to the samples and allowing them to react for 10 min on ice. In previous experiments, the timescale for lysozyme incubation needed for gel samples was screened (SI—Figure S6): No visible difference could be observed for 10 min to 5 h of incubation. The liquid was then removed with a pipette, and the gel samples were washed with 1000 µL of double-distilled water for 3 min by adding the volume with a pipette. After removing the wash solution with a pipette, 500 µL of hybridization buffer (SI—Table S2) containing 50 ng mL^−1^ of the respective probes was added with a pipette, and the samples were incubated overnight at 46 °C, protected from light. The following day, the samples were washed with 1 mL of prewarmed washing buffer (SI—Table S2) by replacing the liquid with a pipette, incubated for 3 min, and then washed again with 1 mL of ice-cooled PBS. Samples were sealed in airtight containers to prevent drying (Section 2.10) and imaged directly.

### 2.9. Buffer

The hybridization buffer was prepared by combining the following components: 180 mL of NaCl (5 M), 20 mL of Tris-HCl (1 M), 500 mL of VE-H_2_O, 300 mL of formamide, and 1 mL of SDS (10% *w*/*v*). The wash buffer was prepared by mixing 20.4 mL of NaCl (5 M), 20 mL of Tris-HCl (1 M), 948.6 mL of VE-H_2_O, 10 mL of EDTA (0.5 M), and 1 mL of SDS (10% *w*/*v*).

### 2.10. Sample Preparation

For sample preparation, liquid cultures were flushed into microscopy slides (15 μ-slide VL 0.4, uncoated, Ibidi, Munich, Germany), while gel cultures were carefully placed on coverslips. To prevent drying, the samples were stored in an airtight environment (SI—Figure S4b). A homemade PLA frame, printed using a 3D printer (X1E, Bambulab, Shenzhen, China), was glued around the slices using liquid glue (UHU Sekundenkleber, UHU GmbH & Co. KG, Bühl, Germany).

Finally, the chamber was sealed with a cover slip and made airtight using dental glue (Addition-curing duplicating silicone, Picodent twinsil, Wipperfürth, Germany). This meticulous preparation ensured that the samples remained in optimal condition and were ready for immediate imaging.

### 2.11. Image Acquisition and Analysis

#### 2.11.1. Inverted Fluorescence Microscopy

Images of liquid cultures were taken with an inverted fluorescence microscope (Ti2-2, Nikon, Tokyo, Japan) equipped with a 40× objective. To measure the FISH probes, the following filter cubes were used: Excitation 472/30, Emission 520/35, and Dichroic Mirror 495 for Alexa488; Excitation 540/20, Emission 575/29, and Dichroic Mirror H559 LP for Cy3; and Excitation 640/30, Emission-690/50, and Dichroic Mirror T660 LPXR for Cy5.

#### 2.11.2. Confocal Microscopy

Image acquisition for the gel slices was conducted using a lightning confocal microscope (SP8, Leica, Wetzlar, Germany) with the software LAS X version 3.5.7.23225 with a 10×/0.45 air objective and HC Pl APO 40×/1.10 water immersion objective. Laser excitation was performed with diode lasers at wavelengths of 405 nm, 488 nm, 552 nm, and 638 nm. The system operated in photon integration mode, using a PMT detector for optimized signal detection. Emission detection was configured to collect wavelengths from 493 to 548 nm, 557 to 643 nm, and 646 to 700 nm for specific fluorophores (e.g., Cy3 and Cy5). The confocal system used a pinhole setting equivalent to 1.00 Airy units for enhanced resolution. Frame accumulation and averaging were set to 1, with unidirectional scanning at a speed of 100 Hz.

#### 2.11.3. Data Analysis

Images were analyzed using the image processing package Fiji (version 1.54f, ImageJ, Open Source). Initially, the images were duplicated, and a background image was generated by applying a Gaussian blur with a width of 15 pixels. This background image was then subtracted from the raw image to enhance the signal-to-noise ratio. Subsequently, the look-up tables (LUTs) were adjusted to optimize contrast and visualization. A region of interest (ROI) measuring 400 by 400 pixels was selected within the area of interest for detailed analysis. One ROI was selected for each gel slice, and three gel slices per sample have been analyzed. A threshold was applied to the image to distinguish particles from the background. The particle analyzer tool of Fiji was then used on the thresholded image, with the analysis being back directed onto the raw data. This process enabled accurate counting of particles within the defined ROI. A detailed description of the specific steps can be found in the Supporting Information (SI—Figures S12 and S13).

## 3. Results and Discussion

### 3.1. Batch Processes in Anaerobic Flasks with a Synthetic Bilayered Biofilm

**Figure 2:**
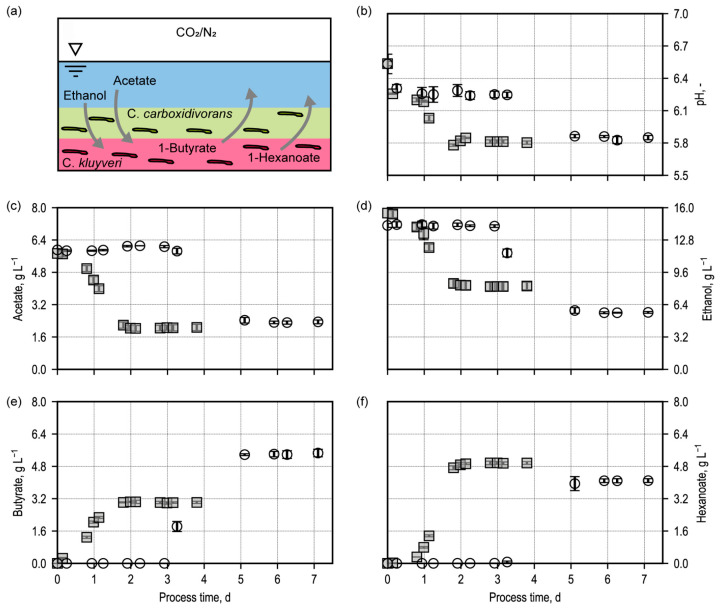
Batch processes in anaerobic flasks with a synthetic bilayered biofilm of *C. carboxidivorans* (top layer) and *C. kluyveri* (bottom layer) in 1.8 % (*w*/*w*) agar hydrogel. (**a**) Scheme of an anaerobic flask with the synthetic bilayered biofilm overlaid with 100 mL of medium operated at 37 °C with an initial CO_2_:N_2_ ratio of 9:1 at a total pressure of 1.5 bar. The concentrations of acetate (**c**), ethanol (**d**), 1-butyrate (**e**), 1-hexanoate (**f**), and the pH (**b**) were measured twice a day. The results of two batch experiments are shown with varying initial cell concentrations: *C. carboxidivorans* and *C. kluyveri* with high initial cell concentrations (3.0 g L^−1^ in the hydrogel) are depicted with squares, and low initial cell concentration (0.3 g L^−1^ in the hydrogel) are shown with circles. Both batch experiments were performed in triplicate.

The batch conversion of ethanol and acetate was studied in anaerobic flasks with two synthetic biofilms at the bottom: *C. kluyveri* was immobilized in the first layer of the hydrogel (average thickness of 4 mm). The second layer had the same thickness containing *C. carboxidivorans* cells. The heterotrophic substrates ethanol and acetate were supplied with the anaerobic fermentation medium above the dual-layered synthetic biofilm. The gas phase in the anaerobic flask had a N_2_:CO_2_ ratio of 1:9 with a total pressure of 1.5 bar (Figure 2a). Ethanol, acetate, and CO_2_ can be consumed by *C. kluyveri* for growth and chain elongation. No growth substrate is available for the immobilized *C. carboxidivorans* cells. Within a batch process time of 7 days, we monitored the pH and analyzed the organic acids and alcohols produced (Figure 2b). To visualize the changes in cell density in the synthetic biofilms of both bacteria, we employed fluorescence in situ hybridization (FISH) after taking biofilm samples.

The immobilized *C. kluyveri* cells become metabolically active after varying lag phases, showing chain elongation by converting ethanol and acetate into 1-butyrate and 1-hexanoate. Concurrent with substrate conversion, the pH decreases from pH 6.8 to approximately pH 5.8 and remains at this value. At low initial cell density (0.3 g L^−1^ in the hydrogel), the lag phase lasts for approximately *3* days, whereas with a tenfold increase in initial cell density (3.0 g L^−1^ in the hydrogel), substrate conversion can already be observed from the beginning of the process. The final acetate concentrations are approximately 2 g L^−1^ for both approaches, while the final ethanol concentrations are lower in the setup with a low initial cell density (~6 g L^−1^) compared to the setup with a high initial cell density (~8.5 g L^−1^). Regarding product concentrations, the setup with a high initial cell density shows a shift in the spectrum towards longer-chain hydrocarbons compared to the setup with a low initial cell density. The final concentrations are approximately 3 g L^−1^ and 5.5 g L^−1^ 1-butyrate and approximately 5 g L^−1^ and 4.2 g L^−1^ 1-hexanoate. The observed metabolic activities can be attributed exclusively to immobilized *C. kluyveri* cells. No suspended cells were detected, as confirmed by the absence of measurable optical density (OD) in the supernatant. This ensures that the metabolic activity observed is solely due to the immobilized cells.

The incomplete consumption of ethanol and acetate can be explained by the drop in pH. *C. kluyveri* remains metabolically active only within a pH range up to approximately pH 5.8. Experiments with the same conditions show that the stoichiometry for approaches with immobilized cells corresponds to that of approaches with suspended cells. However, slight substrate utilization and product formation delays occur when the cells were immobilized. Since it is unclear how many cells are still vital after the immobilization procedure, the initial active dry mass of immobilized cells is most probably reduced, which may explain these delays.

It was clearly shown that *C. kluyveri* is metabolically active within the hydrogel, and the cell count of immobilized cells is, therefore, expected to increase in the hydrogel. However, it remains unclear what happens to the immobilized *C. carboxidivorans* cells during the batch process with a significantly decreasing pH, as neither heterotrophic nor autotrophic substrates required by *C. carboxidivorans* are present. To gain more detailed insights into the behavior and development of the cells within the hydrogel, a qualitative and quantitative determination of the cell concentrations of both microorganisms is required. Therefore, we developed a method based on fluorescence in situ hybridization (FISH) and fluorescence microscopy.

### 3.2. Fluorescence In Situ Hybridization (FISH) for Characterizing Immobilized Bacteria

To be able to track cell growth and distribution of both bacteria, *C. carboxidivorans* and *C. kluyveri*, in our batch processes, we used the FISH labeling method. Characterization and monitoring of the immobilized cells within the synthetic biofilm was achieved with fluorescently labeled probes to bind specific bacterial ribosomal RNA sequences enabling species-specific visualization of the cells. In our study, we employed three probes: EUB338, labeled with Atto 488, which targets a broad range of bacteria to confirm overall cell presence; ClosCarb_1516, labeled with Cy3, designed specifically to identify *Clostridium carboxidovans*; and ClosKluy_1516, labeled with Cy5, to detect *C. kluyveri*. This combination of orthogonal fluorescence markers allowed us to distinguish between the bacterial species within the biofilm and assess their spatial distribution accurately.

The probe EUB338 demonstrated the highest staining efficiency, with approximately 52% of cells successfully stained. Comparatively, the probe ClosCarb exhibited a staining efficiency of approximately 38%, which was slightly higher than that of ClosKluy, measured at 23%. Staining efficiency was determined by calculating the ratio of fluorescently stained bacteria to the total number of bacteria observed under bright field microscopy, in separate gel samples for the two bacterial species (SI—Figures S7 and S8). All probes exhibited high specificity for their respective target bacterial species, with no cross-reactivity observed for non-target bacteria.

### 3.3. Bacterial Distribution and Cluster Formation in Biofilms at Low Initial Cell Concentrations

**Figure 3:**
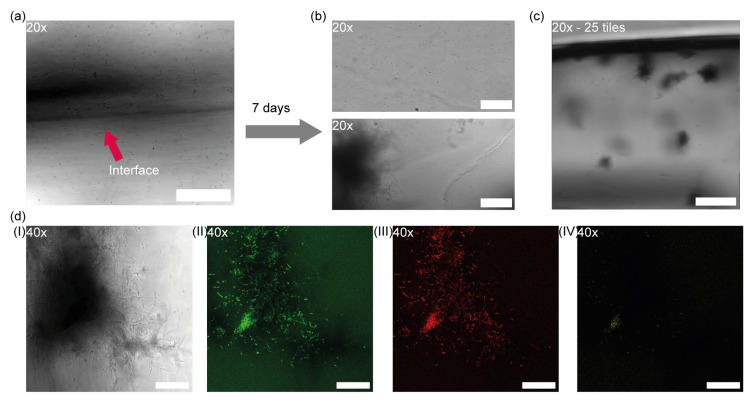
Growth of bacteria immobilized in hydrogel to form a synthetic two-layered biofilm. (**a**) An image of the interface at t = 0 between the two hydrogel slices with the two types of bacteria is shown. Bacteria are homogeneously distributed in the gel with a low initial cell concentration; the bacteria cannot be differentiated. Scalebar: 100 µm. (**b**) After 7 days of cultivation, *C. carboxidivorans* (top) did not grow, and the bacteria are still homogeneously distributed in the gel. *C. kluyveri*, (bottom) on the other hand, grew and formed clusters. Scalebar: 100 µm. (**c**) Clusters of *C. kluyveri* can be observed homogeneously distributed inside the hydrogel. Scalebar: 1000 µm. (**d**) Zoom of one bacteria cluster in the different fluorescent channels for (**I**) brightfield, (**II**) atto488, (**III**) Cy5, and (**IV**) Cy3. *C. kluyveri* can be stained with the general FISH probe EUB338-atto488 (green) and the specific ClosKluy_1516-Cy5 probe (red) but not with ClosCarb_1516-Cy3. Scalebar: 50 µm.

Bacterial cells were initially uniformly distributed throughout the hydrogel matrix. This is illustrated in Figure 3a, which shows the interface between the two layers of the synthetic biofilm. Here, bacteria are dispersed consistently across the gel structure, though it is not possible to visually distinguish between the two bacterial species in this image. The initial homogeneous distribution was intended to represent the starting conditions for subsequent growth and metabolic interaction.

After 7 days of cultivation under anaerobic conditions in flasks, notable differences were observed between the two layers. The upper layer, containing *C. carboxidivorans*, retained its original appearance, with bacterial cells remaining evenly distributed and at a similar concentration to the start of the experiment. The consistent distribution in the upper layer suggests that *C. carboxidivorans* showed no lysis under the conditions provided, without significant cluster formation or migration.

In contrast, the lower layer, which contained *C. kluyveri*, displayed a markedly different morphology after the 7-day period, as shown in Figure 3b. Instead of the original homogeneous distribution, *C. kluyveri* cells form aggregates, visible as distinct clusters that were, nonetheless, evenly distributed throughout the hydrogel layer (Figure 3c). The cells that survived the gel casting process began to grow and form clusters, suggesting that *C. kluyveri*’s growth may be influenced by its immobilization and limited mobility within the synthetic biofilm or by localized environmental conditions within the gel

To confirm the identity of the bacterial cells forming these clusters and to ensure that they corresponded to *C. kluyveri*, FISH probes were used as described in Section 3.2.

Figure 3d presents a close-up view of one such bacterial cluster. Here, intensity signals are displayed across different channels, including the brightfield image (I) and fluorescence channels for Alexa 488 (II), Cy3 (III), and Cy5 (IV). The intensity signals generated by the FISH probes EUB338 (green) and ClosKluy_1516 (red) were clearly detectable, affirming the presence of *C. kluyveri* within the clusters in the hydrogel. This result verifies both the identity of the bacteria forming these clusters and the specificity of the probes used for this experiment.

### 3.4. Growth Dynamics in Synthetic Biofilms at High Initial Cell Concentrations

**Figure 4:**
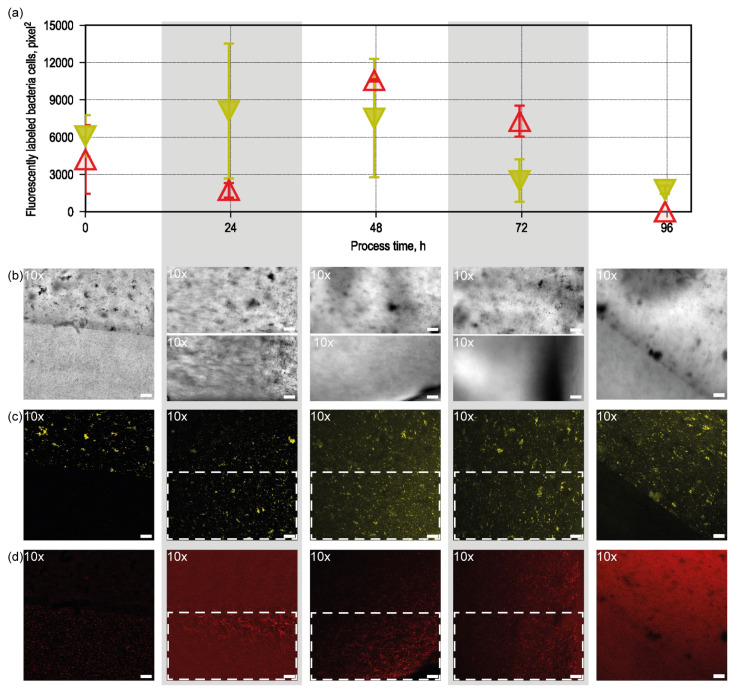
Monitoring changes in cell numbers over time for bacteria with high initial cell concentration (3.0 g L^−1^) in the hydrogel. (**a**) Fluorescently labeled bacteria cells were measured over time. Areas of 400 by 400 pixels were analyzed, and fluorescent areas were summed up to show the amount of bacteria. For each day, triplicates of gel slices were extracted and measured. *C. carboxidivorans* are depicted in yellow, and *C. kluyveri* are depicted in red. After 5 days, *C. kluyveri* cannot be stained anymore, which is probably due to cell death caused by too low pH levels. (**b**) Brightfield showing the *C. carboxidivorans* hydrogel above the *C. kluyveri* hydrogel. (**c**) Cy3 and (**d**) Cy5 images of the gel slices of each layer for days 1 to 5 (from left to right). The dashed rectangles depicts the area which is shown in row (**b**). The interface between the two gel phases can be observed on images for days 1 and 5 as gel slices stayed intact. For days 1–3, the gel phases separated after extraction. Scalebar: 100 µm.

In the experiment with higher initial cell concentrations of 3.0 g L^−1^ in the hydrogel, we applied our FISH labeling method within our synthetic biofilms to characterize growth patterns. The experiment was conducted in eight anaerobic flasks in parallel, with the first flask stopped after 24 h, the second after 48 h, and so on, to collect a sample of the synthetic biofilm for FISH analysis. The corresponding results are displayed in Figure 4, allowing for a comparison of bacterial growth dynamics at different starting concentrations. Interestingly, with this higher initial concentration, we observed that no bacterial clusters formed within the hydrogel matrix, indicating a distinct growth behavior compared to the low-concentration experiment.

Figure 4 provides a detailed analysis of bacterial growth over time in samples with this high initial cell concentration. In Figure 4a, cell counts for both bacterial species over the 4-day cultivation period are displayed. *C. carboxidivorans* (yellow) maintains a stable population throughout, whereas *C. kluyveri* (red) exhibits a significant increase in cell numbers after the initial 48 h, suggesting a delayed but accelerated growth phase for this species. This timeframe corresponds to the period during which an increase in metabolic activity was observed (Figure 2b). Starting from 72 h, the cell count progressively declines, with no cells detectable in the fluorescent channel after 96 h. This is likely due to cell death and subsequent degradation of rRNA in the bacteria, which prevents the binding of FISH probes to their target rRNA sequences.

Figure 4b presents brightfield images of gel slices extracted daily over a 4-day period (further images of the triplicates can be found in SI—Figures S9–S11). The gel slices from days 0 and 4 remained intact, with the interface between the two biofilm layers clearly visible. However, on days 1 through 3, the two layers detached from each other during sample handling, and images could only be captured from individual layers, obscuring the visibility of the interface. Nevertheless, the intact slice observed on day 4 indicates that no substantial changes occurred at the interface. Throughout the hydrogel, bacterial growth appeared uniformly distributed, without localized hotspots or clustering, which further supports the observed difference at higher initial cell concentrations. *C. kluyveri* are homogeneously distributed in the lower layer and *C. carboxidivorans* in the upper layer. The leftmost image shows the situation directly after seeding at day 0. In comparison to Figure 3a, it can be seen that *C. carboxidivorans* forms aggregates at these high seeding densities. In liquid cultures, it was already observed that *C. carboxidivorans* formed aggregates at high cell densities.

Figure 4c,d show fluorescence images capturing the distribution and viability of the bacteria within the hydrogel over time, using Cy3 and Cy5, respectively, for the same image section. As for day 1 to 3, no intact interface could be measured. Figure 4b shows only the lower part of the image sections presented in Figure 4c,d. To quantify cell growth within each layer, 400 × 400-pixel regions of interest were selected from each layer to analyze and measure the cells within these areas. The results of these measurements are presented in Figure 4a. Additionally, no growth of one of the microorganisms into the other layer of the synthetic biofilm could be observed. Figure 4c,d for day 0 and day 4 show that no fluorescence signal could be observed for *C. kluyveri* in the upper part of the hydrogel, and vice versa, no signal for *C. carboxidivorans* could be observed in the lower part of the hydrogel.

These fluorescence images reveal the dynamic distribution of the two bacterial species: *C. kluyveri* exhibits a gradual reduction in signal intensity after 2 days, indicating a decrease in cell viability or population density, while *C. carboxidivorans* maintains consistent fluorescence intensity throughout the first 2 days of the cultivation period, suggesting a stable population and viability over this time. After 2 days, *C. carboxidivorans* also decreases in cell viability. The presumed viability of *C. carboxidivorans* and the assumed decline in cell viability of *C. kluyveri* towards the end of the batch process can be explained by the pH measured throughout the process. It remained within a range where *C. carboxidivorans* is viable [53]. However, for *C. kluyveri*, the pH at the end of the process was too low [54].

## 4. Conclusions

FISH and fluorescence microscopy offered valuable tools for monitoring bacterial distribution and viability within the anaerobic biofilms, enhancing our understanding of growth dynamics in biofilms. The clustering observed in *C. kluyveri* at low initial cell concentrations, as well as the stable distribution at higher concentrations, highlighted the impact of cell density on bacterial behavior. Overall, the results underscore the potential of synthetic biofilms for studying microbial interactions in anaerobic processes and optimizing biofilm-based bioprocesses. The method we developed has broader applications in biofilm research, particularly in examining interactions within synthetic co-culture systems and enhancing microbial process efficiency.

Future experiments could focus on optimizing conditions to promote the metabolic activity of *C. carboxidivorans* within the biofilm, e.g., adding CO to the gas phase for autotrophic growth and product formation (acetate and ethanol). These adjustments could reveal new insights into the spatial behavior and interactions with *C. kluyveri*, deepening our understanding of the metabolic interplay between these species.

Bioprinting of hydrogels with metabolically complementary cells will offer new possibilities for the design of synthetic biofilms with high local resolution [27]. A motivation for the future design of synthetic biofilms is the use of cooperative relationships between selected (and metabolically designed) microorganisms such as mutualism or commensalism, for instance, by cross-feeding, without the necessity of ensuring growth conditions for the cells in the synthetic biofilm [55]. Synthetic biofilms are promising to improve microbial process efficiency, particularly in biochemical production. Future work will aim to refine the biofilm system, explore spatial dynamics of bacterial migration and cooperative relationships, and extend this approach to other microbial consortia.

## Figures and Tables

**Table 1:** FISH probes used for staining *C. carboxidivorans* and *C. kluyveri*. A universal probe that stains both bacteria species and two type-specific probes were used.

Name	Target Organism	Target Molecule	Fluorophores	Sequence
EUB338	Most bacteria	16S rRNA	Alexa488	TTGCTGCCTCCCGTAGGAGT
ClosCarb_1516	*C. carboxi-* *divorans*	23S rRNA	Cy3	AGCCACTCCCCATCACAC
ClosKluy_1516	*C. kluyveri*	23S rRNA	Cy5	GCGGACTCCCCTTCAAAG

## Data Availability

The data presented in this work are available upon request from the corresponding author.

## Supplementary Materials

The following supporting information can be downloaded at https://www.mdpi.com/article/10.3390/microorganisms13020387/s1: Table S1: Composition of the basal medium used for cell cultivation; Table S2: Buffer for FISH staining; Figure S1: Liquid co-culture of Clostridium carboxidivorans and Clostridium kluyveri; Figure S2: Gel samples; Figure S3: PLA stamps for extracting gel cubes; Figure S4: Microtome and airtight chamber for samples; Figure S5: Gel slice and visualization of interface of synthetic biofilm; Figure S6: Incubation with different amounts of lysozyme; Figure S7: Efficiency of FISH probes; Figure S8: Efficiency of FISH probes; Figure S9: Batch cultures of 5 days; Figure S10: Interface overview in different magnifications; Figure S11: Overview of gel slices; Figure S12: Image preparation for particle analysis; Figure S13: Region of interest (ROI) for particle analysis.
