# Supplementary material for: Immobilization and Monitoring of Clostridium carboxidivorans and Clostridium kluyveri in Synthetic Biofilms
Source: Microorganisms. 2025 Feb 10;13(2):387. doi: 10.3390/microorganisms13020387 (PMC11858013; doi:10.3390/microorganisms13020387)
Supplement: Supplementary file 1 [file microorganisms-13-00387-s001.zip › microorganisms-3452283-Supplementary Materials_corrected.pdf]

Supporting Information

Monitoring of immobilized Clostridia with FISH

Josha Herzog[1+], Anna C. Jäkel[2+], Friedrich C. Simmel[2], and Dirk Weuster-Botz[1*]

[1] Technical University of Munich, TUM School of Engineering and Design, Chair of Biochemical Engineering, Boltzmannstraße 15, D-85748 Garching, Germany

[2] Technical University of Munich, TUM School of natural Sciences, Chair of Physics of Synthetic Biosystems, Am Coulombwall 4a, D-85748 Garching, Germany

[+]Shared First-Authors

[*]Corresponding Author: Dirk Weuster-Botz; Technical University of Munich, TUM School of Engineering and Design, Department of Energy and Process Engineering, Chair of Biochemical Engineering, Boltzmannstr. 15, D-85748 Garching, Germany

Tel: +49.89.289.15712

Fax: +49.89.289.15714

Email: dirk.weuster-botz@tum.de

Culture medium

Table S1 – Composition of the basal medium used for cell cultivation

Component	Amount	Dimension
$CaCl_2 \cdot 2\ H_2O$	0.16	$g\ L^{-1}$
$MgSO_4 \cdot 7\ H_2O$	1.23	$g\ L^{-1}$
NH_4Cl	3.00	$g\ L^{-1}$
NaCl	2.40	$g\ L^{-1}$
KCl	0.30	$g\ L^{-1}$
KH_2PO_4	0.30	$g\ L^{-1}$
Nitrilotriacetic acid	0.02	$g\ L^{-1}$
$MnSO_4 \cdot H_2O$	0.01	$g\ L^{-1}$
$NH_4Fe(SO_4)_2 \cdot 6\ H_2O$	0.01	$g\ L^{-1}$
$CoCl_2 \cdot 6\ H_2O$	37.00	$mg\ L^{-1}$
$ZnSO_4 \cdot 7\ H_2O$	36.00	$mg\ L^{-1}$
$CuCl_2 \cdot 2\ H_2O$	0.25	$mg\ L^{-1}$
$NiCl_2 \cdot 6\ H_2O$	0.37	$mg\ L^{-1}$
$Na_2MoO_4 \cdot 2\ H_2O$	0.23	$mg\ L^{-1}$
Na_2SeO_4	0.20	$mg\ L^{-1}$
$Na_2WO_4 \cdot 2\ H_2O$	0.22	$mg\ L^{-1}$
Pyridoxine-HCl	0.12	$mg\ L^{-1}$
Thiamine-HCl	0.06	$mg\ L^{-1}$
Riboflavin	0.05	$mg\ L^{-1}$
D-Ca-Pantothenate	0.05	$mg\ L^{-1}$
Lipoic acid	0.05	$mg\ L^{-1}$
p-Aminobenzoic acid	0.05	$mg\ L^{-1}$
Nicotinic acid	0.05	$mg\ L^{-1}$
Vitamin B12	0.05	$mg\ L^{-1}$
D-Biotin	0.02	$mg\ L^{-1}$
Folic acid	0.02	$mg\ L^{-1}$
MESNA [a)]	0.23	$mg\ L^{-1}$

[a)] (2-Mercaptoethanesulfonic acid sodium salt)

Buffer Used for FISH Staining of Liquid Cultures

Table S2 – Buffer composition

	**Hybridizationbuffer (mL)**	**Washbuffer (mL)**
NaCl (5M)	180	20.4
Tris-HCl (1M)	20	20
VE-H2O	500	948.6
Formamid	300	-
EDTA (0.5M)	-1	10
SDS (10% w/v)	1	1

<u>Protocol for staining bacteria in solution</u>

Cells were harvested, followed by centrifugation for 3 minutes with 13,000 rpm at 4 °C, and the supernatant was discarded. The pellet was dehydrated sequentially with 80% ethanol and 99% ethanol (Art.-Nr. 1HPH.1, Roth), each for 3 minutes. Following dehydra-tion, the samples were centrifuged again for 3 minutes at 13,000 rpm and 4 °C, and the supernatant was discarded. The pellet was then suspended in 400 µL of 1x PBS (pH 7.2 - 7.6, P4417-100TAB, Sigma) and 100 µL of lysozyme solution 10 mg mL-1), Art.-Nr. 8259.2, >45000FIP U/mg, Roth). This suspension was allowed to react on ice for 10 minutes. Sub-sequently, the samples were centrifuged for 5 minutes with 13,000 rpm at 4 °C, and the pellet was washed with double distilled water and then resuspended in 500 µL of hybridization buffer (SI – Table S2) containing 50 ng µL-1 of the respective probes (Main Text Table 1). The mixture was incubated for 3.5 hours at 46 °C, protected from light. After incubation, the samples were centrifuged for 3 minutes at 13,000 rpm, and the supernatant was dis-carded. The pellet was washed with prewarmed wash buffer (SI - Table S2) at 46 °C and then incubated for 20 minutes at 46 °C in the same buffer. Following incubation, the sam-ples were washed with ice-cooled PBS, resuspended in 500 µL of PBS, and imaged direct-ly.

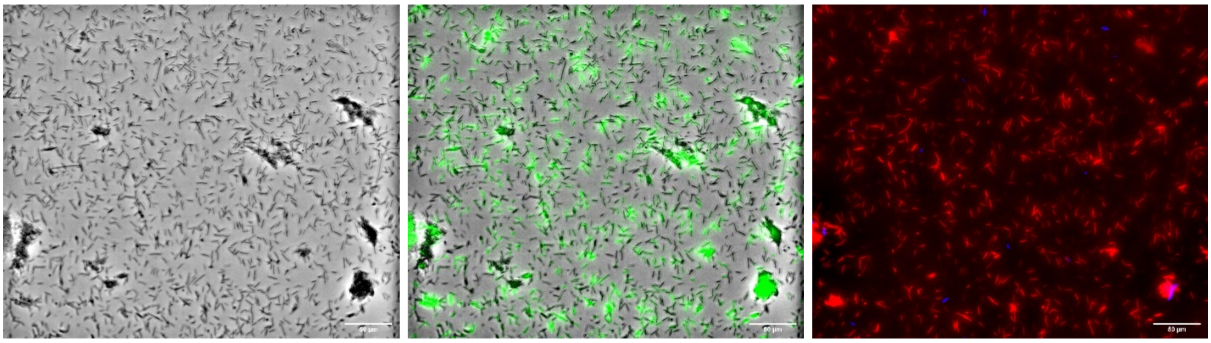

Figure S1 – Liquid co-culture of Clostridium carboxidivorans and Clostridium kluyveri. a) Brightfield of liquid co-culture of Clostridium Carboxidivorans and Clostridium Kluyveri. b) Merged image of brightfield and GFP channel. Probe EUB338_Alexa488 binds to both bacteria cultures. c) Merged image of the fluorescence channels for Cy3 (blue) and Cy5 (red). Liquid Coculture culture of Clostridium Carboxidivorans and Clostridium Kluyveri. Clostdidium Carboxidivorans is only stained with the probe ClosCarb_1516_Cy3 and Clostdridium Kluyveri with the probe ClosKluy_1516_Cy5. The co-culture is dominated by Clostridium Kluyveri.

Preparation of Gel Cubes

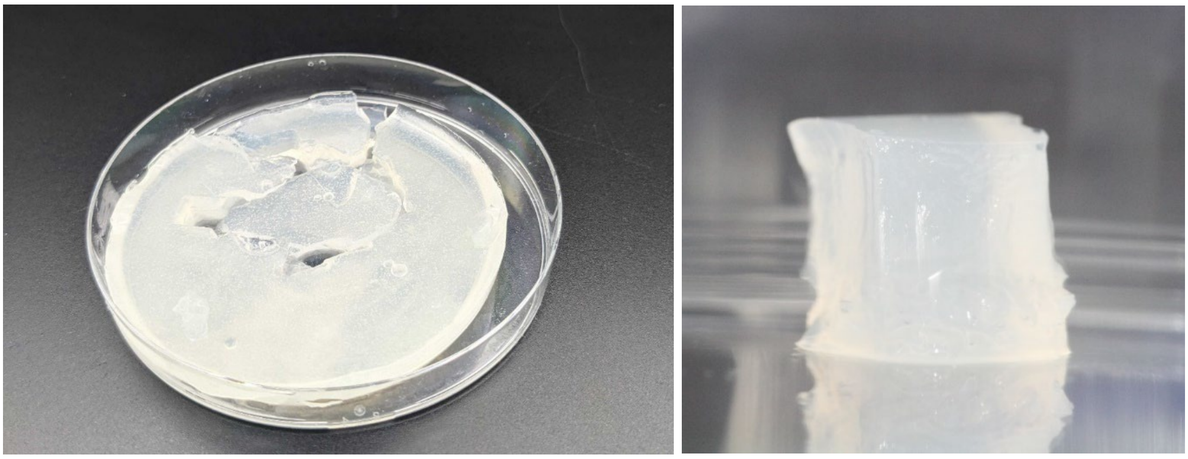

Figure S2 – a) Geldisc extracted from bottle culture for visualization. For experiment analysis the cubes were punched while the discs remained in the bottles to ensure its integrity. b) Gel cube extraced from gel disc with custom-made PLA puncher.

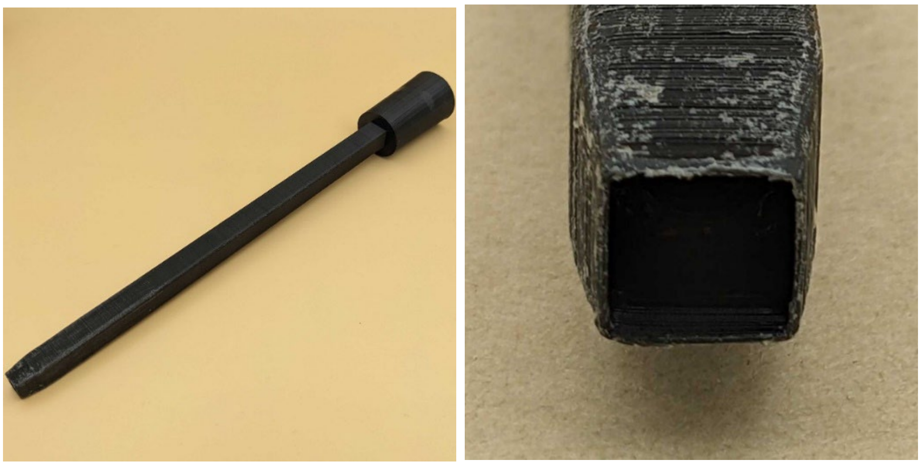

Figure S3 – Custom-made PLA puncher to extract hydrogel cubes from bottle culture experiments. The wall thickness at the tip is 1 mm. A pipetus can be plugged to the other side of the puncher. Through the application of negative pressure, the gel cube can be aspirated, and subsequently, positive pressure can be used to extrude it for further processing.

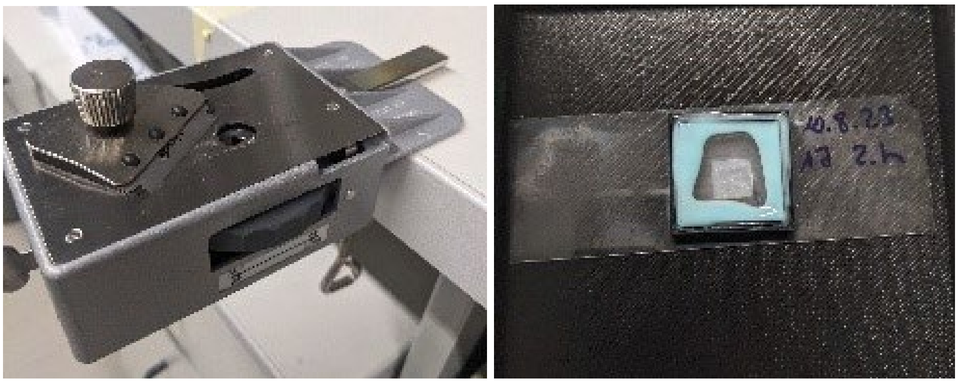

Figure S4 – a) Microtome to slice gel cubes extracted from gel discs. b) Prepared Slice for Imaging.

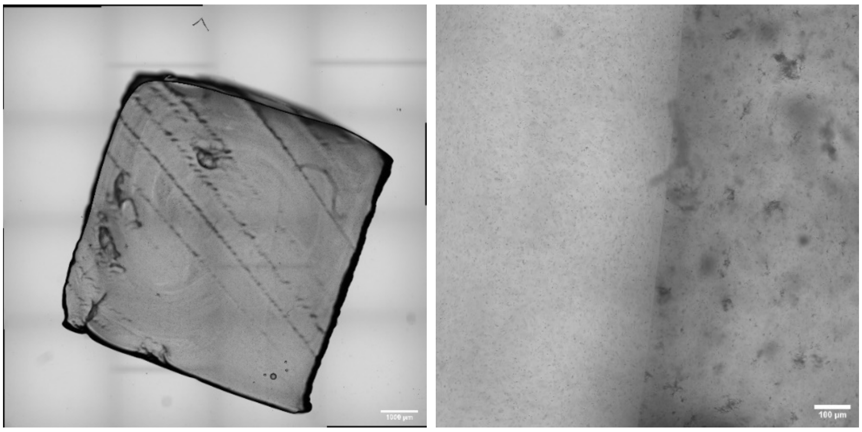

Figure S5 – Cutted Gel Slice. a) Overview of a whole slice. b) Overview of the interface between the two hydrogel layers of right Clostridium Kluyveri and left Clostridium Carboxidivorans.

Test of Incubation Time with Lysozyme

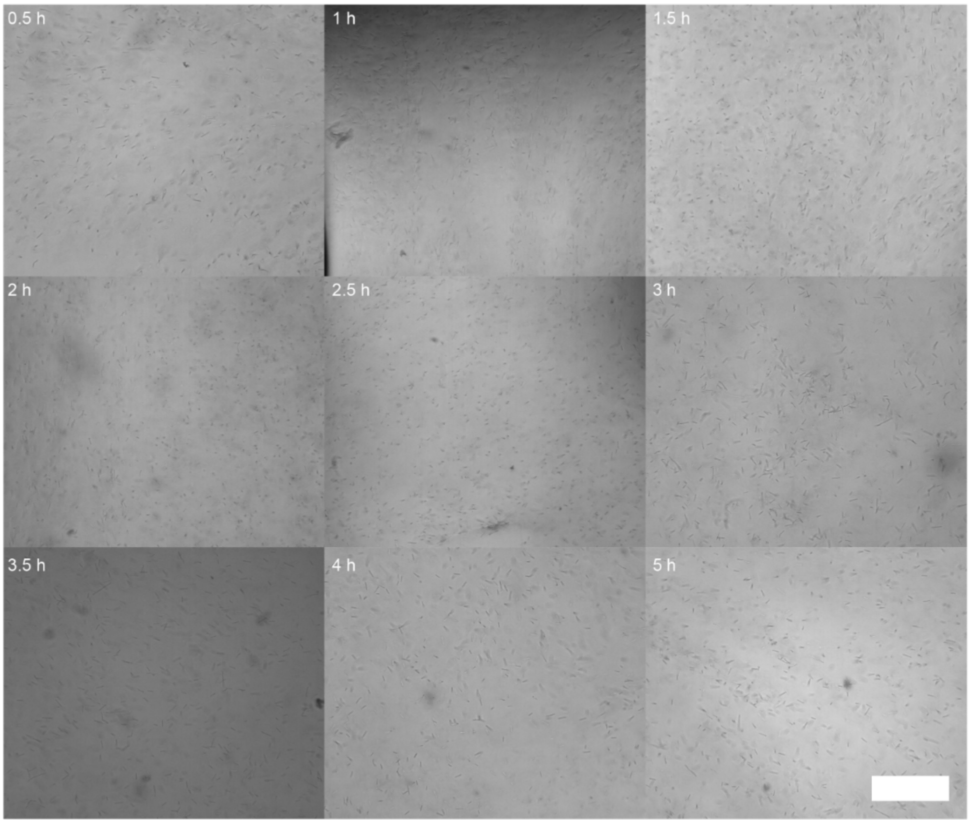

Figure S6 – Incubation of gel slices with different incubation times of lysozyme ranging from 0.5 hours to 5 hours. As the bacteria stay intact no matter for how long they are incubated and good results for intensities of FISH probes were already achieved after 10 minutes of incubation time for lysozyme the shortest incubation was used for further experiments.

Efficiency of Staining Bacteria with FISH probes

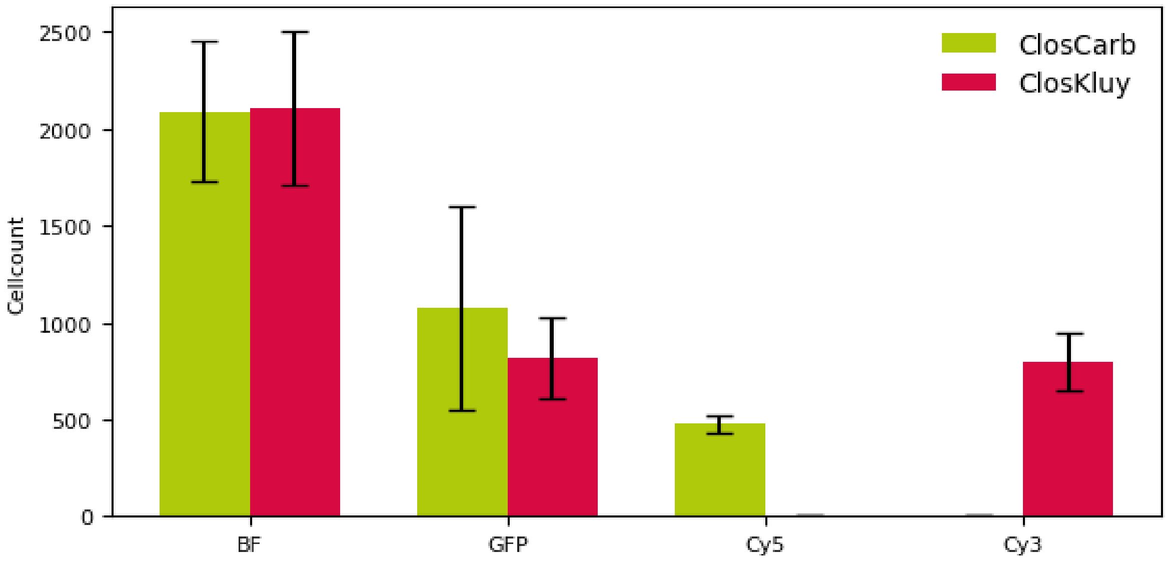

Figure S7 – Counted cells in the different fluorescent channels shows efficiency of staining the bacteria with the FISH probes when cells are cultivated in hydrogel.

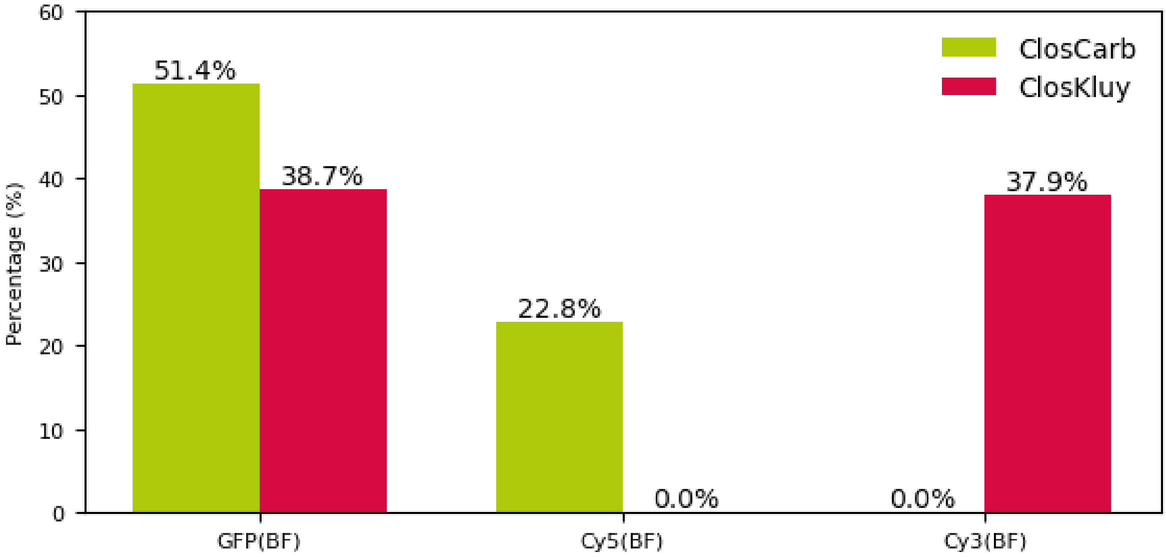

Figure S8 – Percentage of stained bacteria is determined of the counted bacteria in brightfield for the two types of bacteria.

5 Day Bottle Cultivation Experiment

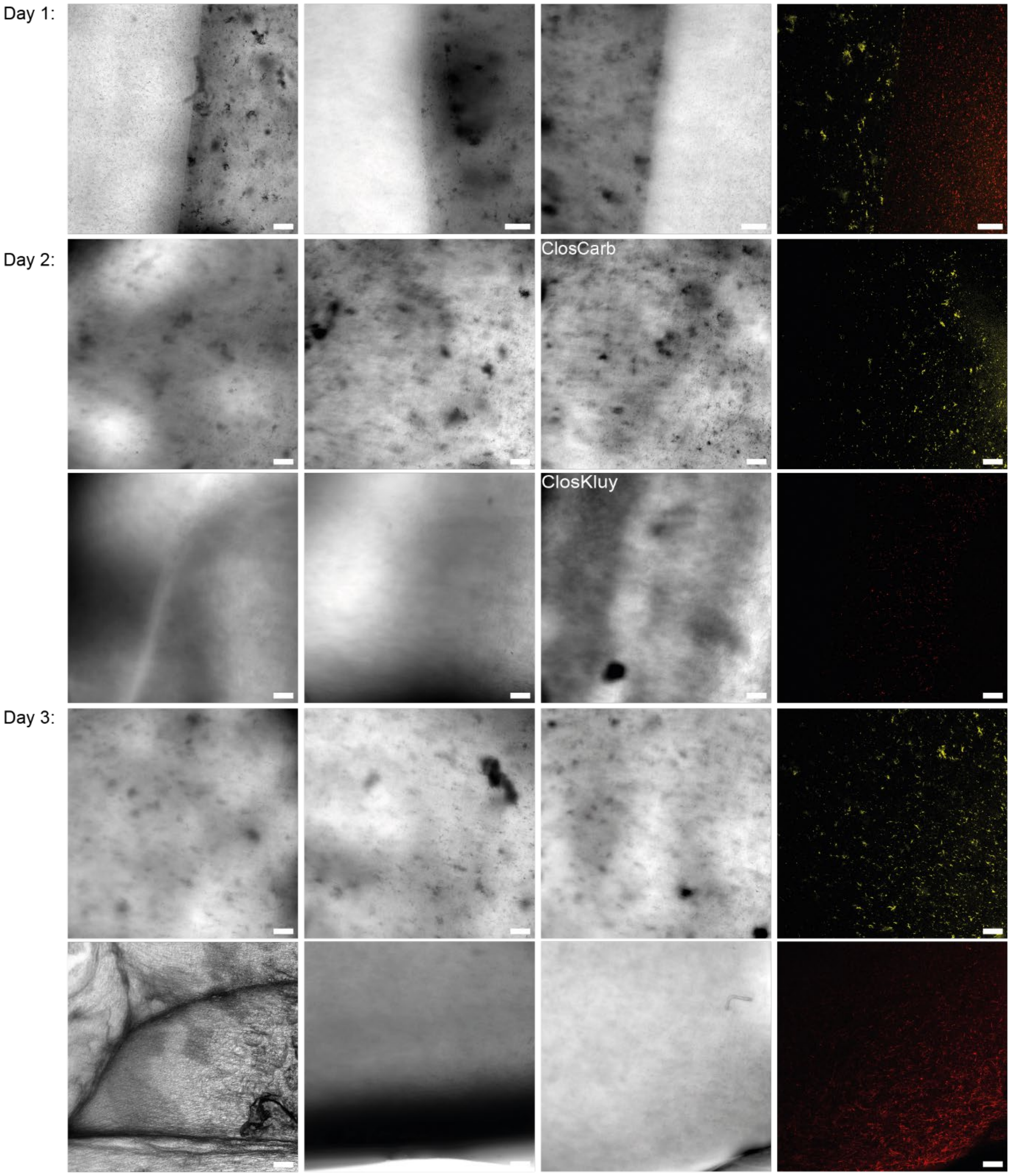

Figure S9 – Brightfield images of Gel cultures from day 1 – 3, triplicates are shown from left to right. The most right column shows the fluorescent channel for triplicate 3. On day 1 the cube could be extracted as a whole with its two layers. On day 2 and 3 the sample cubes could be extracted as whole but while cutting slices the two layers moved apart. Hence the upper row shows results for Clostridium Carboxidivorans and the lower for Clostridium Kluyveri. Bacteria cells are distributed homogeneously in the hydrogel, but the Clostridium Carboxidivorans cells form noticeable clusters from the beginning leading to the hypothesis that they tend to form clusters already in solution. Scalebar 100 µm.

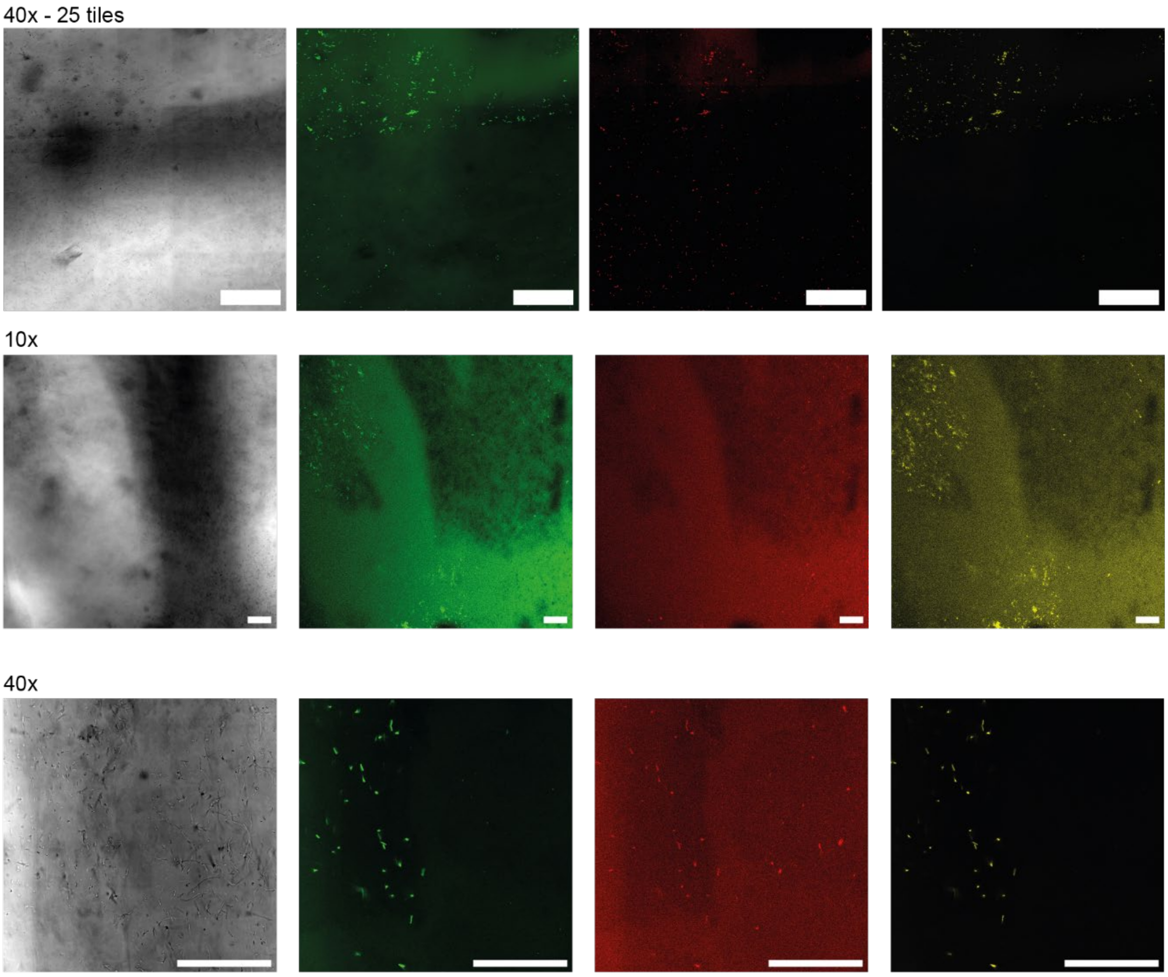

Figure S10 – Images of interface between two hydrogel layers. From left to right: BF, GFP, Cy5, and Cy3. ClosCarb are stained with Cy3 and ClosKluy with Cy5. Scalebar 100 µm.

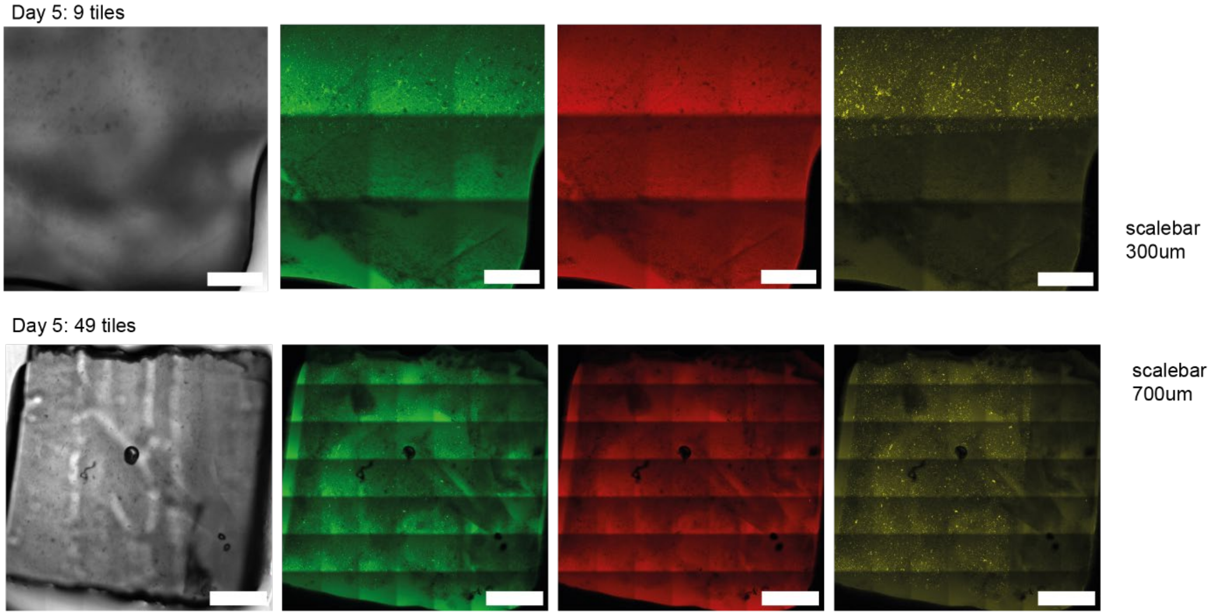

Figure S11 – Overview of 2 samples after 5 days of cultivation. a) scalebar 300 µm and b) scalebar 700µm. ClosKluy are not stainable anymore.

Particle Analysis

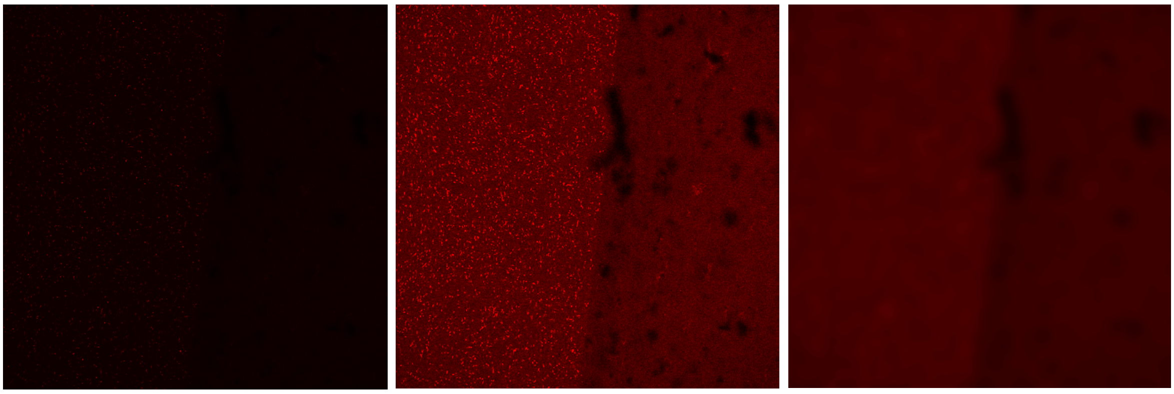

Figure S12 – Raw data of one gel slice in the Cy5 channel. Same image as shown in Figure 17, but LUTs are corrected. Background created with a gaussian blur with 15 pixel width on the image from Figure 18.

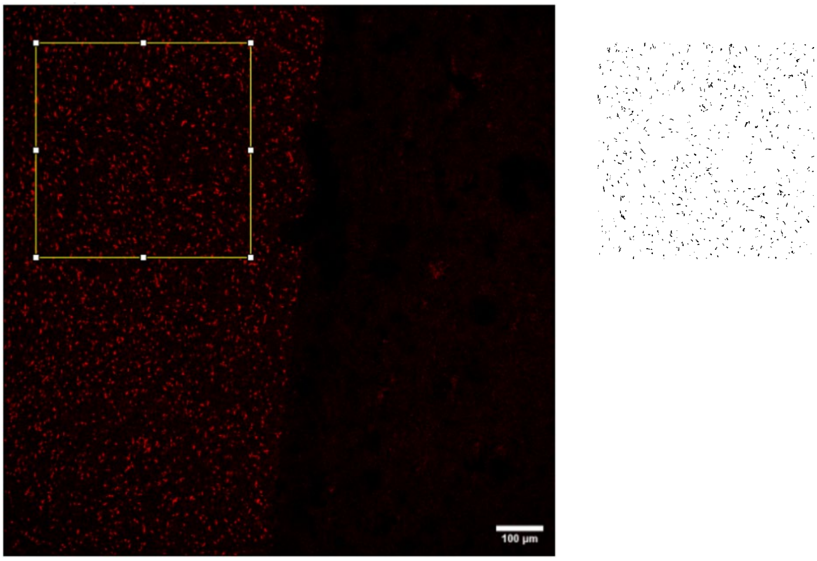

Figure S13 – The created background image was substracted from the LUT corrected image file. A region of interest (ROI) for particle analysis was selected with a size of 400x400 pixels. By applying a threshold on the image from Figure 20 a mask was created to be able to count the particles.

References

[1] Schneider, M.; Bäumler, M.; Lee, N.M.; Weuster-Botz, D.; Ehrenreich, A.; Liebl, W. Monitoring co-cultures of Clostridium carboxidivorans and Clostridium kluyveri by fluorescence in situ hybridization with specific 23S rRNA oligonucleotide probes. Systematic and applied microbiology, 2021, 44, 126271.

[2] Amann, R.I.; Binder, B.J.; Olson, R.J.; Chisholm, S.W.; Devereux, R.; Stahl, D.A. Combination of 16S rRNA-targeted oligonucleotide probes with flow cytometry for analyzing mixed microbial populations. Applied and environmental microbiology, 1990, 56, 1919–1925.
